# Surgery

**Published:** 1879-10

**Authors:** 


*Collaborators:*

DR. H. GRADLE, DR. L. W. CASE, DR. R. PARK,
DR. R. TILLEY.

### SURGERY.

EXTIRPATION OF BOTH OVARIES IN HYSTERO-EPILEPSY—CURE. (By D. E. Welponer, *Wiener Med. Wochenschrift*, Nov. 30, 1879.

Up to date, but two cases of spaying in hystero-epilepsy have been reported; one operated upon by Peaslee with fatal results, and another by Battey, in whom the attacks did not return after the ovariotomy.

The case reported by Dr. Welponer, the assistant at Braun's clinic (Vienna), was an unmarried woman of 36 years, *who had never menstruated.*. Healthy during youth, she showed signs of puberty at 17 years, but until the 20th year, only malaise existed, without any hæmorrhage whatever at the periods corresponding to the menstrual epoch. After the 20th year she suffered from epileptiform attacks occurring at first every four, but later, every three weeks. There had never been any flow of blood, but slight leucorrhœa since the 10th year. The entire list of remedial procedures—including dilatation of the os and local treatment—had previously been exhausted by other physicians without result.

Examination showed a small uterus, with long vaginal portion in slight anteflexion. No anomalies about the vagina or ovaries. A few days before the attack the patient became depressed, slept a good deal, complained of vertigo and cerebral congestion.

These were the customary premonitory symptoms. The attack commenced in the flexion of the hand and fingers, and tonic extension of the extremities, followed at once by loss of consciousness.

Next, the muscles of the neck and trunk entered into violent clonic spasm, whirling the body to and fro as often as 100 times a minute. The attacks recurred 10 to 15 times daily, during a period of 3 to 5 days. Each attack lasted 15 to 30 minutes, and was followed by great prostration.

Of all the numerous therapeutic means employed, compression of the ovaries was the only procedure abbreviating the attack; but it was followed by local pains.

As a last resource, Dr. Braun resorted to ovariotomy according to Hegar's method, with antiseptic precautions.

The surgical history after the operation was complicated by several abscesses, one of which perforated into the vagina, so that complete recovery was protracted until the end of the fifth week.

During the five months she remained under observation after the operation, the epileptic seizures have not returned, although there is still some malaise for some days every month. The patient, however, has gained in weight, and the sexual desire has not diminished since the operation.

The removed ovaries did not present any striking abnormal appearance.

---

WE have received the prospectus of "*The Alienist and Neurologist*," a quarterly journal of practical and scientific psychiatry and neurology. It will be edited by C. H. Hughes, M.D., of St. Louis, Mo., and the initial number will be published about the first of January next. Terms, $5.00 per annum in advance. We know of no one more competent to conduct a journal devoted to the nature, treatment and medico-legal relation of neuropsychic and nervous disease than Dr. Hughes. We have no doubt he will make a journal useful to the general practitioner, and we wish him success.

